# Human Polβ Natural Polymorphic Variants G118V and R149I Affects Substate Binding and Catalysis

**DOI:** 10.3390/ijms24065892

**Published:** 2023-03-20

**Authors:** Olga A. Kladova, Timofey E. Tyugashev, Elena S. Mikushina, Nikita O. Soloviev, Nikita A. Kuznetsov, Daria S. Novopashina, Aleksandra A. Kuznetsova

**Affiliations:** 1 Institute of Chemical Biology and Fundamental Medicine, Siberian Branch of Russian Academy of Sciences, 630090 Novosibirsk, Russia; 2 Department of Natural Sciences, Novosibirsk State University, 630090 Novosibirsk, Russia

**Keywords:** DNA repair, DNA polymerase beta, single-nucleotide polymorphism, enzymatic activity

## Abstract

DNA polymerase β (Polβ) expression is essential for the cell’s response to DNA damage that occurs during natural cellular processes. Polβ is considered the main reparative DNA polymerase, whose role is to fill the DNA gaps arising in the base excision repair pathway. Mutations in Polβ can lead to cancer, neurodegenerative diseases, or premature aging. Many single-nucleotide polymorphisms have been identified in the *POLB* gene, but the consequences of these polymorphisms are not always clear. It is known that some polymorphic variants in the Polβ sequence reduce the efficiency of DNA repair, thereby raising the frequency of mutations in the genome. In the current work, we studied two polymorphic variants (G118V and R149I separately) of human Polβ that affect its DNA-binding region. It was found that each amino acid substitution alters Polβ’s affinity for gapped DNA. Each polymorphic variant also weakens its binding affinity for dATP. The G118V variant was found to greatly affect Polβ’s ability to fill gapped DNA and slowed the catalytic rate as compared to the wild-type enzyme. Thus, these polymorphic variants seem to decrease the ability of Polβ to maintain base excision repair efficiency.

## 1. Introduction

The genetic information encoded in DNA is under constant threat of damage and can be influenced by both external negative factors and internal natural metabolic processes [1,2]. For instance, as a result of aerobic cellular respiration, reactive oxygen species (ROS) are formed [3]. Under the action of ROS, non-bulky oxidative lesions can arise in DNA [4,5,6,7,8,9,10]. Such defects can lead to cell death or malignant transformation [11,12,13,14]. Therefore, during evolution, various systems for restoring the genetic information have developed [15]. One of the most common processes of repair of non-bulky DNA lesions is base excision repair (BER). BER is a process involving a set of enzymes that recognize a damaged base, remove it, introduce a strand break near the resulting apurinic/apyrimidinic site (AP site), incorporate an appropriate nucleotide, and ligate the break [16,17,18,19,20,21,22,23,24].

Polβ expression is essential for the cell’s response to DNA damage that occurs during natural cellular processes [25]. Polβ is thought to be the main reparative DNA polymerase filling DNA gaps with complementary dNMPs [26]. Accordingly, mutations in the *POLB* gene can influence Polβ functioning and can lead to cancers [27], neurodegenerative diseases [28,29], or premature aging [30,31,32]. It is known that functionally deficient Polβ mutants have a low efficiency of DNA repair, thus raising the frequency of mutations in the genome [25,33,34,35,36,37,38,39]. Studies indicate that up to 30% of analyzed human tumors express Polβ polymorphic variants [27]. The detected single-nucleotide polymorphisms (SNPs) are not concentrated in any specific region of the protein and are located in all subdomains of Polβ. It is known that mutations that affect the dRP-lyase or polymerase activity of Polβ [39,40] reduce the effectiveness of BER and cause hypersensitivity to alkylating or oxidizing agents. Previously, we have analyzed known human Polβ polymorphisms [41]. This analysis suggests that some polymorphisms can lead to substitutions of functionally significant amino acid residues and therefore affect the catalytic activity of the enzyme and the accuracy of insertion of nucleotides. Nevertheless, some polymorphic variants of Polβ contain amino acid substitutions that are far from the polymerase’s active site but affect the catalytic stage and have been found in various types of cancer.

**Figure 1:**
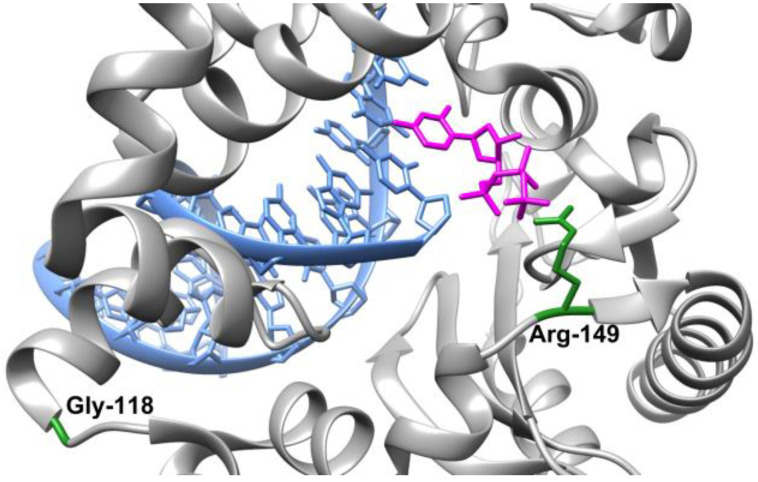
Location of selected amino acid residues (green) of Polβ (grey) in complex with 1nt-gap–containing DNA (blue) and an incoming deoxyribonucleoside triphosphate (pink). Protein Data Bank (PDB) ID: 5UPG.

According to the NCBI database, there are more than 12 thousand known nucleotide substitutions in the *POLB* gene, most of which occur in introns. In the coding region, 349 variants (nucleotide substitutions) have been detected that change the class of amino acids and hence can alter the protein’s function. Previously [41], with bioinformatic approaches, 22 polymorphisms have been revealed causing an amino acid substitution and having a high probability of affecting Polβ functioning. In the present study, we obtained two variants of Polβ in the form of recombinant proteins corresponding to such SNPs: G118V (rs764967314) and R149I (rs779188078). The aim was to experimentally examine their enzymatic properties in comparison with the wild-type (WT) enzyme. An analysis of X-ray data [42,43] revealed that both Gly118 and Arg149 are located in loop regions of the protein’s globule, namely between α7 and α8 helixes and between α11 and α12 helixes, respectively. Nonetheless, Arg149 takes part in the coordination of the γ-phosphate (Pγ) of an incoming nucleotide (Figure 1). Moreover, it has been shown that another natural polymorphic variant, G118Q, occurs in esophageal cancer tissues [44]. Therefore, we tested the two substitutions for their influence on the structure of Polβ, on the main stages of its catalytic cycle (formation of a binary complex with DNA and assembly of a ternary complex with DNA and incoming dNTP), and on the catalytic efficiency of dNMP incorporation.

## 2. Results and Discussion

### 2.1. The Influence of the Amino Acid Substitutions on Enzyme Folding

**Figure 2:**
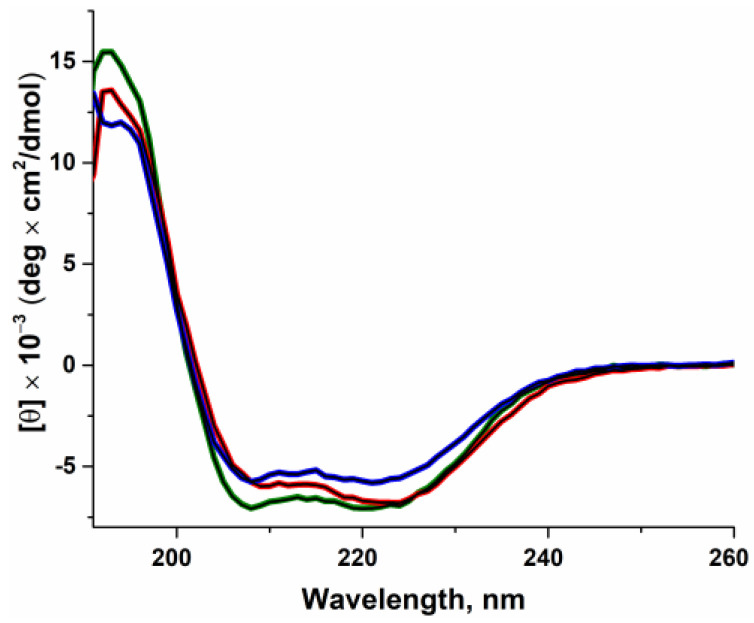
CD spectra of WT Polβ (green) and of variants G118V (red) and R149I (blue). The simulation results are the black curve.

The shapes of the circular dichroism (CD) spectra were similar among all the studied proteins (Figure 2). Polβ is among the proteins with predominance of α-helices in their structure, and the calculated percentages of α-helices in the studied proteins are shown in Table 1. From the obtained data on the shape of the CD spectra and on similar contents of α-helices in the structure, it can be concluded that the amino acid substitutions G118V and R149I do not cause a global change in the secondary structure of the protein.

### 2.2. Molecular Dynamics Simulations of Free-State Enzymes

All known crystal structures of Polβ in the absence of a substrate have a wide-open conformation subtly different between individual structures [45,46,47]. In such a wide-open state, the centers of mass of an N-terminal 8 kDa domain and of the C-terminal “fingers” domain are ~50 Å apart, which is twice as far as in either the open protein–DNA binary complex or the closed protein–DNA–dNTP ternary complex.

**Figure 3:**
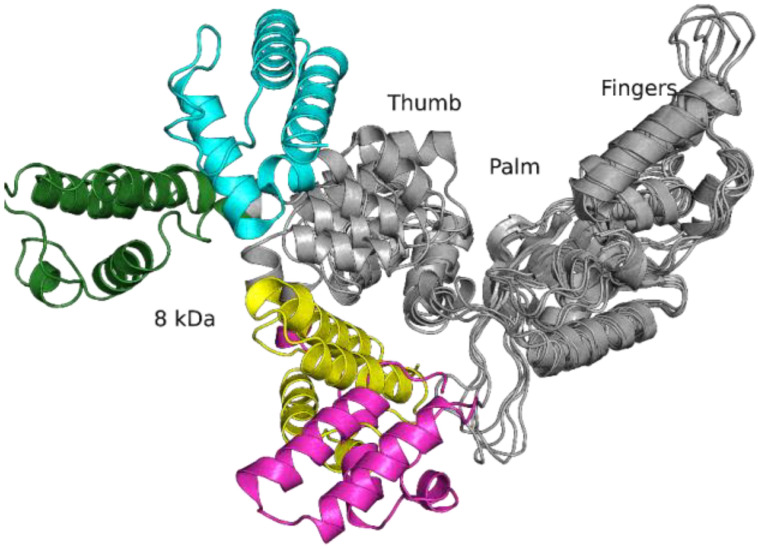
An overlay of rat Polβ apo-enzyme crystal structure 3UXN (cyan) and representative snapshots of unbiased MD simulation for human WT Polβ (green) and its variants G118V (magenta) and R149I (yellow). The parts of mutant variants structure that overlaps with WT rat and human Polβ structure are colored in grey.

It was found that during the molecular dynamics (MD) simulations, the WT enzyme and both SNP variants shift from their crystal structure conformations, owing to flexible hinges in the Polβ thumb domain. The WT apo-enzyme extends its wide-open state with an increase in the distance between the 8 kDa domain and the fingers domain up to 70 Å, but in both mutant enzymes, the 8 kDa domain and thumb domain adopt more compact conformations, drawing closer to the palm domain. In this compact conformational state, N-terminal amino acid residues form multiple transient hydrogen bonds and hydrophobic contacts with residues Gly144–Ile149 of the hinge region and Asp246–Tyr250, which are a part of the loop in the “fingers” domain (Figure 3).

Model structures suggest that in the free state, the Polβ SNP variants undergo a disturbance of equilibrium toward a more compact structure, which probably will reduce the enzymatic activity as compared to the WT enzyme because of the emergence of bulky hydrophobic amino acid residues next to the mobile sites of the enzyme. Despite the changes in the arrangement of domains, the models did not reveal considerable alterations of the secondary structure of the proteins, consistent with the results of CD spectroscopy (Figure 2).

### 2.3. DNA-Binding Affinity of the Polβ Variants

**Figure 4:**
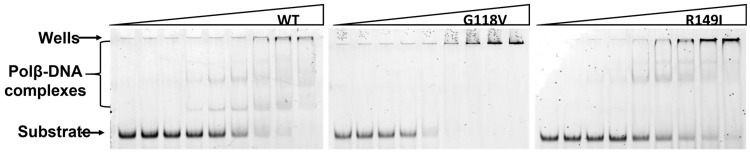
The EMSA of DNA binding by WT Polβ and by polymorphic variants G118V and R149I. The experiments were conducted using serial dilutions of the enzymes. The concentration range was from 2.83 μM to 22 nM for WT Polβ, from 16.4 μM to 130 nM for the G118V variant, and from 11.6 μM to 91 nM for the R149I variant. [DNA substrate] = 50 nM.

**Figure 5:**
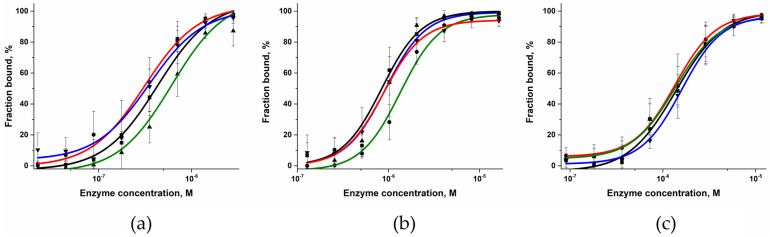
Efficiency of binding of WT Polβ (**a**), G118V (**b**), or R149I (**c**) to DNA substrates containing different nucleotides opposite the gap (black: Gap_A (■), red: Gap_T (●), green: Gap_G (▲), and blue: Gap_C (▼)).

The ability of the Polβ SNP variants to form a complex with DNA containing a 1 nt gap was tested under the same conditions in an electrophoretic mobility shift assay (EMSA) (Figure 4). During this analysis, the formation of several complexes with different mobility in the gel was recorded. Of note, the formation of several complexes with different gel mobility in the EMSA has also been reported earlier [48,49,50]. It is possible that Polβ forms complexes with DNA in some fixed intermediate states between the well-known open and closed conformations, and these intermediate states possess different electrophoretic mobility. The analysis of the gel images allowed us to determine the dependence of the DNA-bound fraction on the enzyme concentration (Figure 5), and this dependence was utilized to calculate the dissociation constants *K*_d_ using Equation (1) (Table 2). Unexpectedly, the dissociation constants for the wild-type protein were in the 0.33–0.59 μM range, that is, ~tenfold higher than expected for the *K*_d_ values of Polβ (60 nM [51] 30 nM [48], 5 nM [33]). However, such a discrepancy of *K*_d_ values most likely was achieved owing to the different length and type of the DNA substrates used in these studies as well as the difference in experimental conditions (pH and ionic strength of the buffer solution). Nevertheless, a comparison of *K*_d_ values for WT enzyme and SNP variants in the same experimental conditions allowed us to elucidate the effect of amino acid substitutions.

The obtained dissociation constants *K*_d_ revealed that WT Polβ has similar abilities to bind a gapped DNA substrate in cases of A, T, and C placed at the position opposite to the gap, but *K*_d_ is ~1.5-fold higher for G. Notably, this finding was also made about both SNP variants, suggesting that guanosine placed at the position opposite to the gap slightly destabilizes the enzyme–DNA complexes.

It was found that the dissociation constants *K*_d_ for the variants G118V and R149I are ~2- and threefold higher than this constant of the WT enzyme (Table 2). This result could indicate moderate destabilization of the enzyme–DNA complex after a substitution of amino acid residue Val118 or Ile149.

### 2.4. MD Simulations of the Binary Open-State Enzyme–DNA Complex

**Figure 6:**
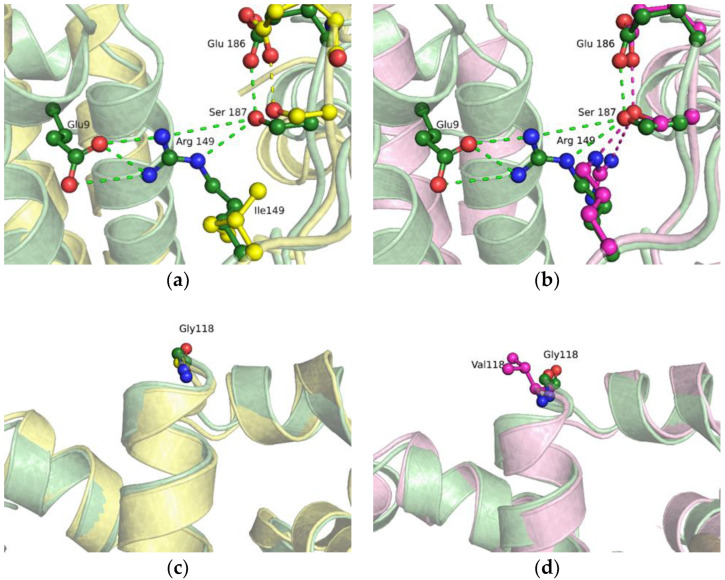
An overlay of WT Polβ (green), G118V (magenta), and R149I (yellow) snapshots of the Arg149 region (**a**,**b**) or Gly118 region (**c**,**d**) in the binary enzyme–DNA complex. Hydrogen bonds are presented as dashed lines.

To elucidate the molecular consequences of substitutions G118V and R149I for the DNA-binding ability of Polβ, MD simulations of the binary open-state enzyme–DNA complex were performed (Figure 6). During the MD simulations, the open-state complex models underwent little change compared to the original crystal structure, with the backbone’s root mean square deviation (RMSD) staying under 0.3 nm. In the WT enzyme, the sidechain of Arg149 maintained hydrogen bonds with the backbone carbonyl and with the sidechain hydroxyl oxygen atoms of the Ser187 residue. Substitution R149I resulted in a loss of these contacts (Figure 6a) but did not alter the orientation and other contacts of Ser187. Additionally, the Arg149 sidechain was able to directly interact with the sidechains of the N-terminal amino acid residues, mostly Glu9, with a hydrogen bond between these residues in existence for 28% of the total simulation time for the complex of the WT protein. In the case of the G118V substitution, which is far from position Arg149, the lifetime of a contact between residues Arg149 and Glu9 diminished to only 9% relative to the WT (Figure 6b). Although this state is not the most common in the MD trajectory, it results in the N-terminal domain’s drawing closer to the palm domain and thereby can affect the enzyme–DNA complex formation. Indeed, when binding to DNA, Arg149 is involved in the transition of the enzyme from an open- to a closed-state structure while forming contacts with the N-terminus of the enzyme. Moreover, R149I and even G118V, which affected the Arg149 region, did not lead to substantial changes in the region of the Gly118 residue (Figure 6c,d).

### 2.5. MD Simulations of the Ternary Closed-State Enzyme–DNA–dNTP Complex

**Figure 7:**
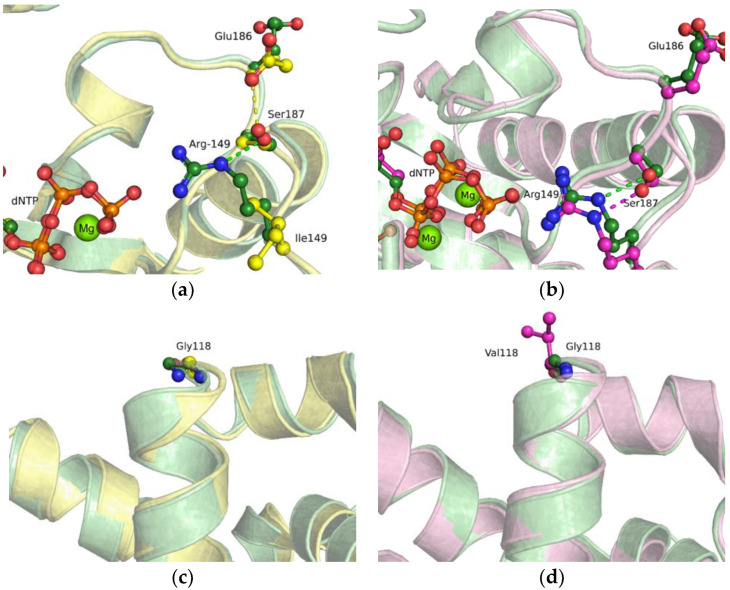
An overlay of WT Polβ (green), G118V (magenta), and R149I (yellow) snapshots of the Arg149 region (**a**,**b**) or Gly118 region (**c**,**d**) in the ternary enzyme–DNA–dNTP complex. Hydrogen bonds are presented as dashed lines.

This complex remained stable under the simulation conditions, with RMSD no more than 2 Å as compared to the initial structure and minor differences in the MD trajectory between the two SNP variants. Nonetheless, in the case of the R149I substitution, the Glu186 sidechain (which coordinates the Ser187 residue) was preferentially oriented inward, similarly to the crystal structures and the model of the binary complex (Figure 7a), in contrast to the G118V model (Figure 7b), where Glu186 mostly maintained an outward orientation as in the crystal structures of the ternary complex. The loss of this coordination contact in the G118V variant could affect both the efficiency of dNTP binding and the correct placement of dNTP in the active site for the catalytic reaction. Again, neither substitution R149I nor substitution G118V led to major changes in the region of the Gly118 residue (Figure 7c,d).

### 2.6. Polymerase Activity of the Two Polβ Variants

**Figure 8:**
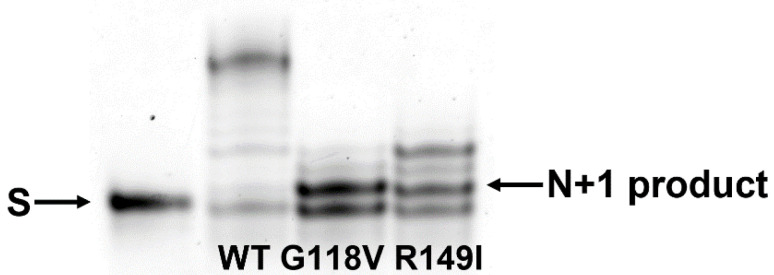
DNA elongation by Polβ variants via strand-displacement synthesis. [DNA substrate] = 0.5 μM, [enzyme] = 1.0 μM, and [dNTP] = 10 μM. Reaction time was 1 min. “S” denotes the initial 19 nt FAM-labeled primer strand. “N + 1 product” represents the DNA product after gap-filling incorporation of a nucleotide resulting in the formation of a nick-containing 20 nt structure. Upper products are results of strand-displacement DNA synthesis.

To investigate the effect of the amino acid substitutions on the polymerase activity of Polβ, a gapped DNA substrate containing a FAM label was used. After gap-filling incorporation of a nucleotide resulting in the formation of a nick-containing 20 nt structure, WT Polβ was able to perform strand-displacement DNA synthesis (Figure 8). Unexpectedly, both variants G118V and R149I SNP had a much lower activity during the incorporation of the first nucleotide into the gapped DNA substrate and during the subsequent elongation of the primer by strand-displacement DNA synthesis (Figure 8). Indeed, Polβ G118V yielded a barely noticeable accumulation of DNA products that had more than 1 additional nucleotide incorporated. By contrast, in the case of Polβ R149I, an accumulation of DNA products containing up to three additional nucleotides was detectable.

**Figure 9:**
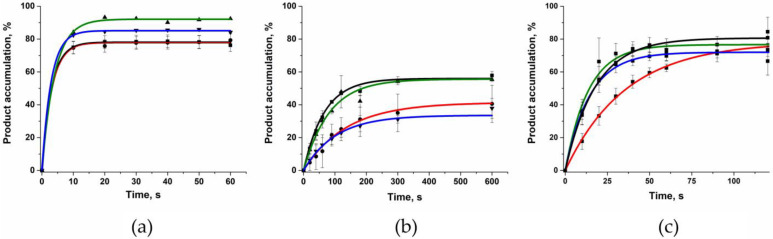
The dependence of the increase in the concentration of the reaction product on time. (**a**) WT Polβ, (**b**) G118V, and (**c**) R149I. The product accumulation for substrate Gap_A (■) is shown as the black curve, for Gap_T (●) as the red curve, for Gap_G (▲) as the green curve, and for Gap_C (▼) as the blue curve. [DNA substrate] = 0.5 μM, [enzyme] = 0.5 μM, [dNTP] = 5 μM.

To estimate the rate constants of single-nucleotide incorporation into the gapped DNA substrate, the kinetics of product accumulation was analyzed next (Figure 9). In this set of experiments, only one type of complementary dNTP was added to the reaction mixture. The obtained kinetic traces of product accumulation with different nucleotides opposite the gap allowed us to calculate the observed rate constants using Equation (2) (Table 3). An analysis of the rate constants indicated that the incorporation of dCTP into substrate Gap_G proceeded slightly more slowly than did the incorporation of the other nucleotides. Probably, this effect is based on the destabilization of the enzyme–DNA complex in the case of substrate Gap_G, as revealed by the EMSA (Table 2).

It turned out that the observed rate constants for the G118V variant were at least 20-fold lower than those of WT Polβ, whereas for the R149I variant, the reduction was only three- to fivefold (Table 3). It should be noted that in the case of variant R149I, the decrease in the observed rate constants had the same order of magnitude as the reduction in the DNA-binding ability (Table 2). On the other hand, Polβ G118V manifested the lowest product accumulation level, indicating that this substitution exerts combined effects: it leads not only to a decrease in the DNA-binding ability but also to significant disturbances of the catalytic complex, thereby preventing the efficient incorporation of dNTP, as suggested by the MD simulations. Taken together, the results indicate that the G118V substitution influences both the stage of formation of the complex with DNA and the stage of incorporation of dNTP into the synthesized DNA strand. In turn, the dNTP-incorporation step depends both on the efficiency of dNTP binding to the enzyme and on the efficiency of the catalytic reaction.

### 2.7. Conformational Changes of DNA and Measurement of dNTP-Binding Affinity

To estimate the dNTP-binding ability of the SNP variants, the conformational dynamics of the Pol_Gap_2-aPu DNA duplex, which contains a single-nucleotide gap and a 2-aminopurine reporter residue, were recorded using the stopped-flow method. For this purpose, a 1.0 μM solution of the enzyme was mixed with a 0.5 μM solution of the DNA substrate containing various concentrations of dATP.

**Figure 10:**
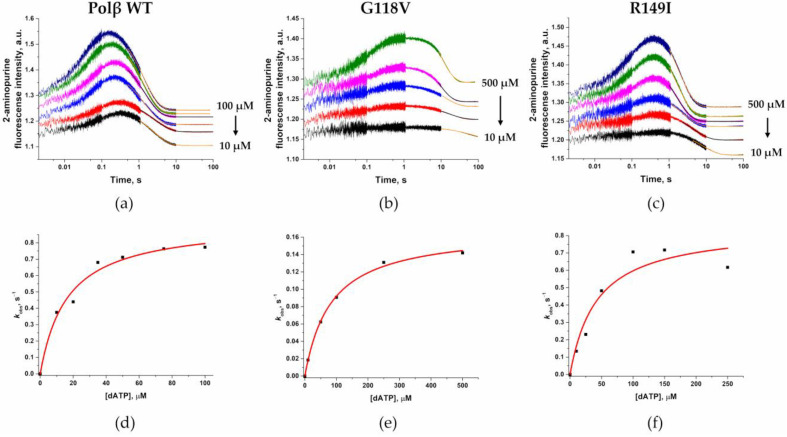
Changes in fluorescence intensity of the 2-aminopurine residue (present in the DNA substrate) upon interaction with WT Polβ (**a**), Gly118Val (**b**), or Arg149Ile (**c**) at various concentrations of dATP. Changes in dATP concentrations are shown in the figures. [Enzyme] = 1.0 μM, [DNA substrate] = 0.5 μM. Solid orange or black lines represent fitted part of curves. The dependence of the calculated values of the observed rate constants on the dATP concentration is shown in panels (**d**), (**e**), and (**f**) for WT Polβ, Gly118Val, and Arg149Ile, respectively.

Incorporation of dNTP into the DNA substrate resulted in biphasic changes in the fluorescence intensity of the 2-aminopurine residue (Figure 10). It is known [52,53] that the phase of increasing 2-aminopurine fluorescence intensity corresponds to two stages: formation of the enzyme–DNA complex and subsequent formation of a ternary open-state complex with dNTP. At the 100 μM dATP concentration, the growth phase of 2-aminopurine fluorescence intensity ended by time point 0.2, 1.5, or 0.5 s for WT Polβ, G118V, and R149I, respectively. The difference in the change in the 2-aminopurine signal indicates differences in the rates of the reactions and in the efficiency of the formation of the ternary complex.

The second slow phase of the decrease in the fluorescence intensity of 2-aminopurine ended at ~10 s for the WT enzyme and for the R149I variant and by time point ~50 s in the case of the G118V variant. This change in the fluorescence intensity signal of 2-aminopurine has been reported to correlate with the following steps: assembly of a ternary closed-state complex, changes in the conformation of this complex to reach a catalytically competent state, the chemical step of nucleotide transfer to DNA, and product accumulation [52,53]. The exponential fitting of this phase enabled us to calculate the observed rate constants according to Equation (3). The dependence of the observed rate constants on the dATP concentration was fitted to Equation (4) to estimate the rate constants of the chemical step (*k*_pol_) and the observed dissociation constant *K*_d, app (dATP)_ (Table 4).

It was found that both mutants have a higher observed dissociation constant *K*_d_, _app (dATP)_ by 5.2- and 2.6-fold for G118V and R149I, respectively, indicating an influence of these amino acid substitutions on the stage of binding of the enzyme–DNA binary complex to dATP. The rate constant of the chemical step *k*_pol_ was almost the same between the WT enzyme and the R149I variant, whereas for the G118V variant, *k*_pol_ was 5.5-fold lower.

These findings indicate that the observed decrease in the overall polymerase activity seen in the R149I variant can be due to a weaker DNA-binding affinity and weaker dNTP-binding affinity but not to changes in *k*_pol_. By contrast, the G118V amino acid substitution led to a decrease in all tested parameters of the enzymatic process by affecting every stage of the polymerization cycle.

## 3. Materials and Methods

### 3.1. Site-Directed Mutagenesis and Protein Purification

Mutations G118V and R149I within the Polβ coding sequence were generated with site-directed mutagenesis. Primer sequences are presented in Table 5. For expression of the recombinant proteins, 2 L of *Escherichia coli* strain Rosetta II (DE3) culture in LB broth (SERVA Electrophoresis GmbH, Heidelberg, Germany) carrying the pET28-c Polβ construct was grown at 50 mg/mL kanamycin and 37 °C until absorbance at 600 nm (OD_600_) reached 0.5; WT Polβ’s and the two mutants’ expression was induced overnight with 0.1 mM IPTG. The collected cells were lysed twice using a French press. The resulting lysate was centrifuged at 40,000× *g* and 4 °C for 40 min. A Q-Sepharose column (Cytiva, Washington, DC, USA) was equilibrated with a buffer composed of 20 mM HEPES pH 7.8 and 200 mM NaCl. Then, the lysate was loaded onto the column, which was next washed with the same buffer at a flow rate of 2 mL/min. The NaCl and imidazole concentrations in the protein-containing fraction were adjusted to 500 and 15 mM, respectively, and this solution was added to 2 mL of Ni-NTA resin (Thermo Fisher Scientific, Waltham, MA, USA) and incubated with stirring for 1.5 h (4 °C). The slurry was carefully packed into the column and washed with 10 mL of a buffer consisting of 20 mM HEPES, 15 mM imidazole, and 500 mM NaCl, after which the column was washed with 10 mL of a buffer composed of 20 mM HEPES, 90 mM imidazole, and 500 mM NaCl. Then, the column was washed with 7 mL of a buffer consisting of 20 mM HEPES, 440 mM imidazole, and 500 mM NaCl, and the eluate was collected. A protein-containing fraction was transferred into a 10 mL dialysis bag pre-washed with dialysis buffer (20% of glycerol, 20 mm HEPES, 150 mm NaCl) and dialyzed overnight. The enzyme concentration was determined using the Bradford method. The enzymes’ solutions were supplemented with 50% of glycerol and stored at −20 °C.

### 3.2. Oligodeoxyribonucleotides

The sequences of the oligodeoxyribonucleotides used in this work are shown in Table 6. FAM-labeled substrates containing a 1 nt gap were obtained by mixing equimolar amounts of three DNA strands: FAM_Pol19, Pol_36_N, and Pol16. 2-Aminopurine–labeled substrates were obtained by mixing equimolar amounts of Pol16, Pol19, and Pol36_N_aPu. The DNA substrates ware annealed for 5 min at 93 °C and allowed to cool to room temperature.

### 3.3. Circular Dichroism (CD) Spectroscopy

CD spectra were recorded on a Jasco J-600 spectropolarimeter (Jasco, Tokyo, Japan) at room temperature in quartz cells with a 0.1 mm light path length. The concentration of Polβ in the device cell was 20 μM. The experiments were carried out in a buffer consisting of 50 mM Tris-HCl pH 7.5, 50 mM KCl, 1.0 mM EDTA, and 5.0 mM MgCl_2_. The spectra were recorded at bandwidth 1.0 nm and wavelength from 190 to 260 nm. The scans were accumulated and automatically averaged. To describe the spectra, we used an online tool for the fitting and simulation of CD spectra of proteins (http://lucianoabriata.altervista.org/jsinscience/cd/cd3.html, accessed on 28 January 2022) [54].

### 3.4. Molecular Dynamics (MD) Simulations

Models of a binary open-state Polβ-DNA complex and a ternary closed-state Polβ-DNA-dNTP complex were constructed based on the respective crystal structures of human Polβ-DNA [55,56,57], with DNA adjusted to depict the truncated experimental oligonucleotide sequences. The structure of the human Polβ apo-enzyme was homologically modeled using Modeller with Chimera interface based on the crystal structure of rat Polβ [45,58,59]. The simulation setup and simulations were performed using GROMACS [60]. The starting structures were solvated and neutralized in a dodecahedral PBC box using TIP3P model water and 50 mM of KCl JC ions [61,62]. The AMBER 14SB force field with OL15 corrections was chosen to describe the protein and the DNA primer [63,64,65,66]. The nucleoside triphosphate parameters were obtained following an established approach using R.E.D. Server [67,68]. Magnesium ions were simulated as octahedral dummy models [69] to preserve the active-site geometry [70]. The cutoff for nonbonded interactions was set to 1.0 nm, with long-range electrostatic interactions analyzed using the PME method [71,72]. Covalent bonds involving hydrogen atoms were constrained using the LINCS algorithm [73]. Steepest-descent energy minimization was followed by 1 ns NVT and NPT equilibrations with solute heavy atoms restrained, using the Bussi thermostat and Parrinello–Rahman barostat [74,75]. Flat-bottomed distance restraints were applied to heavy atoms involved in hydrogen bonds of the terminal base pairs to prevent fraying of the truncated DNA. Postequilibration unrestrained MD simulations were run for 100 ns in triplicate for binary and ternary complexes and for the apo-enzyme, with one model for each mutant complex extended up to 300 ns. Trajectory processing was performed using the integrated GROMACS toolset. Images were generated in the open-source version of PyMOL Viewer. The free energy of complex formation was evaluated through the MMPBSA approach with the gmx_MMPBSA tool [76,77,78].

### 3.5. Electrophoretic Mobility Shift Assay (EMSA)

A 10% native Tris/borate/EDTA (TBE) polyacrylamide gel (75:1) was pre-run for 1 h at 200 V in 0.5× TBE buffer. Recombinant WT Polβ and mutants were serially diluted in a buffer (50 mM Tris-HCl pH 7.5, 50 mM KCl, 5 mM MgCl_2_, 1 mM EDTA, 1 mM DTT, and 7% of glycerol), mixed with 5 μL of a DNA substrate to attain a final DNA concentration of 50 nM, and incubated at room temperature for 15 min. The resultant samples were loaded onto the pre-run gel without any loading buffer. The gels were subjected to electrophoresis at 200 V for 40 min and were scanned using a Versa Doc imager (Bio-Rad Laboratories, Hercules, CA, USA). The gel images were quantified in the Gel-Pro 4 analyzer software (Media Cybernetics, Rockville, MD, USA). The dissociation constant *K*_d_ of each Polβ–DNA complex was calculated according to the equation

Fraction bound (%) = F_u_ + (F_b_ − F_u_)/(1 + (*K*_d_/[Polβ])^h^) (1)

where h is Hill’s coefficient, F_u_ denotes a background contribution, and F_b_ represents the maximal intensity of the complex.

### 3.6. Polymerase Reaction Analysis

To determine the activity of Polβ and its mutants, a solution of a 1-nt-gapped DNA substrate and a complementary dNTP was mixed with an enzyme solution. In the final reaction mixture, the concentrations of the enzyme and gapped DNA were 0.5 µM, and the dNTP concentration was 5 µM. The reaction was carried out at 37 °C in a buffer composed of 50 mM Tris-HCl pH 7.5, 50 mM KCl, 1 mM EDTA, 5 mM MgCl_2_, 1 mM DTT, and 7% of glycerol. Aliquots (10 μL) were taken from the reaction mixture at several time points. The enzymatic reaction was stopped with the addition of an equal volume of a stop solution (7.5 M urea, 25 mM EDTA, 0.1% of xylene cyanole, 0.1% of bromophenol blue). The obtained samples were applied to a denaturing 15% polyacrylamide gel. The resulting gel was visualized in the Versa Doc gel-documenting system (Bio-Rad Laboratories, Hercules, CA, USA). The degree of substrate transformation was determined as the ratio of the peak areas of the product to the sum of the peak areas of the product and of the peak of the initial substrate in the Gel-Pro 4 analyzer software (Media Cybernetics, Rockville, MD, USA). The obtained data were fitted to the equation

[Product] = A × (1 − exp(−*k*_obs_ × t)) (2)

where A is the amplitude, *k*_obs_ is the rate constant, and t is the reaction time.

### 3.7. Stopped-Flow Fluorescence Measurements

The conformational dynamics of a 2-aminopurine–labeled 1-nt-gap-containing DNA substrate were studied using the stopped-flow technique with the detection of a fluorescence signal generated by 2-aminopurine using an SX.20 stopped-flow spectrometer (Applied Photophysics, Leatherhead, UK). A wavelength λ_ex_ = 310 nm was employed for excitation, and emission was analyzed at λ_em_ > 370 nm (Schott filter OG-370). The concentration of Polβ was 1 μM, that of the DNA substrate was 0.5 μM, and the concentration of dATP was varied from 10 to 500 μM. The concentrations of reactants reported are those in the reaction chamber after mixing. All stopped-flow fluorescence measurements were carried out at 37 °C in a buffer consisting of 50 mM Tris-HCl pH 7.5, 50 mM KCl, 5 mM MgCl_2_, 1 mM EDTA, 1 mM DTT, and 7% of glycerol. A slow phase of a decrease in the 2-aminopurine fluorescence intensity on the kinetic curves helped us to calculate the observed rate constant, as shown previously [53]. The data obtained in the fluorescence stopped-flow kinetic assays were fitted using the following exponential equation using the OriginLab software 2015 (9.2) (OriginLab Corp., Northampton, MA, USA):

F = F_0_ + F_1_ × exp(−*k*_obs_ × t) (3)

where F is the observed 2-aminopurine fluorescence intensity, F_0_ is the background fluorescence, F_1_ is a fluorescence parameter, and *k*_obs_ denotes the observed rate constant.

A graph of the dependence of the observed rate constants on the dATP concentration was built to estimate a catalytic rate constant, *k*_pol_, and an apparent dissociation constant of dATP (*K*_d, app (dATP)_) via fitting to this equation:

*k*_obs_ = *k*_pol_ × [dATP]/(*K*_d, app_ + [dATP]) (4)

## 4. Conclusions

The expression of Polβ is essential for the cell’s response to the emergence of DNA damage that may occur during natural cellular processes. It is known that functionally deficient Polβ mutants can have a low efficiency of DNA repair, thereby possibly increasing the occurrence of mutations in the genome. SNPs in the DNA polymerase β gene can have various consequences. Polymorphic variants of Polβ may contain substitutions of amino acid residues important for maintaining the native structure of the enzyme, providing contacts with DNA, influencing the catalytic activities of the enzyme, and playing a role in the correct incorporation of an incoming dNTP into the DNA.

In this work, we studied the effects of previously uninvestigated polymorphic variants of DNA polymerase β: amino acid substitution G118V or R149I in the DNA-binding region. The main stages of the enzymatic process such as binding of the enzyme to DNA, the formation of the ternary enzyme–DNA–dNTP complex, and the catalytic incorporation of dNTP into the synthesized DNA strand were analyzed with MD simulations and experimental approaches. It was demonstrated that each mutation, G118V and R149I, affects almost every analyzed stage of the Polβ enzymatic cycle. It was found that the G118V substitution greatly slows the catalytic stage of Polβ and weakens its affinity for gapped DNA. The R149I substitution does not influence the catalytic constant *k*_pol_ but affects both DNA binding and dNTP binding. Taken together, the results indicate that the two natural polymorphic variants in the Polβ sequence strongly affect the enzymatic activity and thereby may alter the efficiency of BER and the frequency of mutations in the genome.

## Figures and Tables

**Table 1:** Percentages of α-helices in the studied proteins.

WT Polβ	Polβ G118V	Polβ R149I
78.6 ± 15.7	78.4 ± 16.7	64.7 ± 12.3

**Table 2:** Dissociation constants *K*_d_ (μM) of the enzyme–DNA complex.

Substrate	WT Polβ	Polβ G118V *	Polβ R149I
Gap_A	0.38 ± 0.02	0.85 ± 0.09	1.37 ± 0.07
Gap_T	0.33 ± 0.03	0.88 ± 0.07	1.35 ± 0.06
Gap_G	0.59 ± 0.07	1.3 ± 0.1	1.56 ± 0.05
Gap_C	0.38 ± 0.03	0.93 ± 0.07	1.36 ± 0.09

* Hill’s coefficient h = 2 was fixed in these calculations.

**Table 3:** Observed rate constants *k*_obs_ (s^−1^) of gap-filling incorporation of various dNTPs into the DNA strand.

Substrate	WT Polβ *	Polβ G118V	Polβ R149I
Gap_A	0.33 ± 0.03	0.015 ± 0.001	0.056 ± 0.001
Gap_T	0.32 ± 0.04	0.007 ± 0.001	0.028 ± 0.001
Gap_G	0.25 ± 0.02	0.012 ± 0.001	0.073 ± 0.008
Gap_C	0.34 ± 0.02	0.010 ± 0.001	0.069 ± 0.003

* Due to rapid product accumulation, the calculated *k*_obs_ are not smaller than indicated values.

**Table 4:** The rate constant of the chemical step (*k*_pol_) and observed dissociation constant *K*_d, app (dATP)_.

	WT Polβ	Polβ G118V	Polβ R149I
*k*_pol_, s^−1^	0.93 ± 0.05	0.17 ± 0.01	0.9 ± 0.1
*K*_d_, _app (dATP),_ μM	16 ± 3	83 ± 6	42 ± 18

**Table 5:** Primer sequences used for site-directed mutagenesis.

Polβ Mutant	Primer Sequence
G118V	Forward: 5′-GCAAGGAAGTTTGTAGATGAAGTAATTAAAACACTAGAAGATCTCAG-3′ Reverse: 5′-CTGAGATCTTCTAGTGTTTTAATTACTTCATCTACAAACTTCCTTGC-3′
R149I	Forward: 5′-GGGGACTTTGAAAAAATAATTCCTCGTGAAGAGATGTTACAAATG-3′ Reverse: 5′-CATTTGTAACATCTCTTCACGAGGAATTATTTTTTCAAAGTCCCC-3′

**Table 6:** Sequences of oligonucleotides.

Name	Sequence
Pol16	5′-TAGTCACCTCAATCCA-3′
FAM_Pol19	FAM 5′-GCCTCGCAGCGGTCCAACC-3′
Pol19	5′-GCCTCGCAGCGGTCCAACC-3′
Pol36_N	5′-TGGATTGAGGTGACTANGGTTGGACGGCTGCGAGGC-3′
Pol36_N_aPu	5′-TGGATTGAGGTGACTXNGGTTGGACGGCTGCGAGGC-3′

FAM is 6-carboxyfluorescein, X is 2-aminopurine, and N is A, T, G, or C.

## Data Availability

Raw experimental data are available from O.A.K. and A.A.K. upon request.
